# The Ash2l SDI Domain Is Required to Maintain the Stability and Binding of DPY30

**DOI:** 10.3390/cells11091450

**Published:** 2022-04-25

**Authors:** Mengjie Ma, Jiafeng Zhou, Zhihua Ma, Hanxue Chen, Liang Li, Lin Hou, Bin Yin, Boqin Qiang, Pengcheng Shu, Xiaozhong Peng

**Affiliations:** 1 The State Key Laboratory of Medical Molecular Biology, Neuroscience Center, Medical Primate Research Center and Department of Molecular Biology and Biochemistry, Institute of Basic Medical Sciences, Chinese Academy of Medical Sciences, School of Basic Medicine Peking Union Medical College, Beijing 100005, China; joeyma97@outlook.com (M.M.); alanzhoujf@outlook.com (J.Z.); zhihuama1991@163.com (Z.M.); chenhx7072@outlook.com (H.C.); liang.li@ucsf.edu (L.L.); houlin@ibms.pumc.edu.cn (L.H.); yinbin_pumc@outlook.com (B.Y.); chiangbq@imicams.ac.cn (B.Q.); 2 Chinese Institute for Brain Research, Beijing 102206, China; 3 Institute of Laboratory Animal Science, Chinese Academy of Medical Sciences and Peking Union Medical College, Beijing 100021, China

**Keywords:** COMPASS, Ash2l, Dpy30, protein degradation

## Abstract

ASH2L and DPY30 are important for the assembly and catalytic activity of the complex associated with SET1 (COMPASS), which catalyzes histone methylation and regulates gene expression. However, the regulations among COMPASS components are not fully understood. Here, we leveraged a mouse model and cell lines to observe the outcome of Ash2l depletion and found a significant decrease in DPY30. Analyzing ASH2L ChIP-seq and RNA-seq data excluded transcriptional and translational regulation of ASH2L to DPY30. The decrease in DPY30 was further attributed to the degradation via the ubiquitin-mediated proteasomal pathway. We also verified that three amino acids in the ASH2L Sdc1 DPY30 interaction (SDI) domain are essential for the recognition and binding of DPY30. Lastly, we unexpectedly observed that overexpression of DPY30 in Ash2l-depleted cells rescued the decrease in Ccnd1 and the abnormal cell cycle, which indicates that DPY30 can participate in other complexes to regulate gene expression. Overall, our results, for the first time, reveal that the existence of DPY30 relies on the binding with ASH2L, with degradation of DPY30 via the ubiquitin-proteasome system, and they further indicate that the function of DPY30 can be independent of ASH2L.

## 1. Introduction

Gene expression regulation is a complex yet finely controlled process in eukaryotes, in which histone modifications play a critical role and contribute to spatial and temporal pattens of gene expression, further influencing almost all aspects of cell physiology and pathology. One of the most widespread and well-known histone modifications is histone H3 lysine 4 methylation (H3K4me), which is catalyzed predominantly by a family of evolutionarily conserved methyltransferases containing SET domains and associated with actively transcribed genomic loci. The first histone H3 lysine 4 (H3K4) methylase SET1 was identified in *Saccharomyces cerevisiae*, and it functions in complexes with other protein partners, named the complex associated with SET1 (COMPASS) [1]. In mammalian cells, the yeast methyltransferase SET1 has six homologous proteins, namely, SET1A, SET1B, MLL1, MLL2, MLL3, and MLL4, also known as MLL family proteins, to recognize the H3K4 tail of target enhancers or promoters by its functional SET domain and to catalyze different numbers of methyl groups to the lysine of histones [2]. Each methyltransferase can form different types of COMPASS with other proteins to exercise unique functionalities, and each complex contains an MLL family methyltransferases and four core proteins—WRAD (or WARD), which are WD repeat containing protein 5 (WDR5), Retinoblastoma Binding Protein 5 (RbBP5), Absent-Small-Homeotic-2-Like protein (ASH2L), and Dumpy-30 (DPY30).

The WRAD subunits are also evolutionarily conserved and can assemble in the absence of the MLL subunit [3]. This subcomplex is necessary for the normal assembly and catalytic function of COMPASS [4,5,6,7,8], as the enzymatic activities of methyltransferases are quite low without the combination of WRAD [3,6]. Interestingly, biochemical reconstitution experiments in vitro and structural analysis have revealed that the WRAD complex has an intrinsic binding pattern. Cosgrove and colleagues analyzed the pairwise interactions of the MLL1 core complex and found the interaction direction to be MLL1–WDR5–RbBP5–ASH2L–DPY30 [9]. In addition, several independent studies revealed the precise regions of each subunit allowing the assembly of the WRAD complex. In particular, ASH2L has different functional domains: the SPRY domain for binding RBBP5, and the SDI domain for binding DPY30 [10]. Notably, DPY30 participates in the complex through binding three amino-acid residues (L513/L517/V520) in the SDI domain of ASH2L [11,12], and a special stoichiometric ratio (2:1) exists between DPY30 and ASH2L [13]. However, it is not fully understood the outcome after the disruption of the WRAD subcomplex, such as the stability of each subunit and the influence on biological processes such as the cell cycle.

H3K4 methylation modification has always been recognized as an active hallmark of gene expression in proliferating cells [14,15,16,17]. Dysregulation of H3K4 methylation and the core members of the COMPASS complex underlie diverse pathologies, especially neurological developmental disorders and neoplastic pathogenesis [18,19]. For example, deficiencies in Mll1, Mll2, and Mll3 are closely correlated with Wiedemann–Steiner syndrome [20], Kabuki syndrome [21], and Kleefstra syndrome [22], respectively. Conditional knockout of different COMPASS family members in different tissues has demonstrated the important functionality of these subunits in development [23]. Interestingly, ASH2L and DPY30 have been reported to be related to changes in cell proliferation and the cell cycle [24,25,26,27,28]. ASH2L-cKO NSCs showed cell-cycle arrest in the G1 phase [23], and SET1-deleted cells also displayed delayed entry into the S phase [24]. In pancreas development, the cell cycle was arrested in the G1 phase when the expression of DPY30 was disrupted [28]. DPY30 regulates the expression of cell-cycle-related genes to facilitate the proliferation and differentiation of hematopoietic progenitor cells (HPCs) [25]. Soon after, ASH2L was revealed to play the same role in HPC, and ASH2L disrupted cell arrest in the G2 phase [27]. DPY30 is highly expressed in epithelial ovarian cancer cells, and the disruption of DPY30 cells results in arrest in the G0/G1 phase [29]. Cell-cycle dysregulation is a hallmark of many kinds of cancers, and cell-cycle regulation is also important for the development of a creature. Thus, it is necessary to explain the abnormal cell cycle in abnormal development.

Herein, we pinpointed the dependency of DPY30 on ASH2L via in vivo and in vitro experiments and verified that the stability of DPY30 relies on binding with ASH2L. We also verified the three special-amino acid residues of the ASH2L SDI domain to which DPY30-D/D binds, as this connection is crucial for the stability of DPY30. If the binding sites of these two proteins are destroyed, DPY30 degradation is accelerated by the ubiquitin-proteasome pathway. In addition, we previously found that the expression of the G1 phase protein Cyclin D1 was dramatically downregulated in the ASH2L-cKO neocortex. Current in vitro experiments proved that the expression of Ccnd1 was decreased both transcriptionally and translationally when ASH2L is knocked down, whereas overexpression of DPY30 could partly rescue Ccnd1 expression.

## 2. Materials and Methods

### 2.1. Animals

D6-Cre; Ash2l fl/fl mice were used as described previously [23]. Ash2l-cKO mice were bred in the Animal Center of Peking Union Medical College. All animal-related operations and experiments followed the rules of the Experimental Animal Regulations (China Science and Technology Commission Order No. 2) and the Institutional Animal Care and Use Committee at the Academy of Medical Sciences and Peking Union Medical College.

### 2.2. Cell Culture

HEK293T, HeLa, and N1E-115 cell lines were grown in Dulbecco’s modified Eagle’s medium (DMEM) with 10% fetal bovine serum (FBS) and 1% penicillin–streptomycin and heated at 37 °C in 5% CO_2_. MG132 (Sigma, St. Louis, MO, USA, #M7449), Chloroquine (Sigma, St. Louis, MO, USA, #C6628), actinomycin D (Sigma, St. Louis, MO, USA, #SBR00013), and cycloheximide (Millipore, Burlington, NJ, USA, #239763) were used to treat cells.

### 2.3. Tissue Section and Immunofluorescence

Fetal mouse brains were fixed in 4% PFA–PBS for 24 h and then switched to 25% sucrose for 24–48 h to dehydrate. The brain was embedded into O.C.T. and immediately placed in a −80 °C refrigerator. The embedded brain was sliced by a cryogenic tissue slicer.

Tissue slices were warmed to 50 °C for 30 min, washed with 1× PBS for 5 min, and then incubated with 5% sheep serum (1× PBS, 0.3% Triton X–100) for 30 min to block antigen sites. Primary antibodies were diluted with buffer solution (1 × PBS, 0.3% Triton X-100) at a ratio of 1:300–1:500. Tissue slices were incubated with primary antibodies in a handmade wet box overnight at 4 °C. The tissue slices were washed with 1× PBS three times, each time for 5 min. The secondary antibodies were diluted with buffer solution at a ratio of 1:800 and incubated with tissue slices for 2 h at room temperature. After washing three times, the tissue slices were treated with DAPI and sealed with coverslips. The tissue slices were observed and photographed by Zeiss (LMS780).

### 2.4. Plasmid Construction

Plasmids were constructed by conventional gene cloning protocols and transfected with Lipo3000 reagent. After 10–12 h, the culture medium was changed, and cells were harvested after 36–48 h. siRNA was transfected with NTERFERin, and the culture medium was changed if necessary. Cells were harvested at approximately 72 h. When plasmids and siRNA co-transfection occurred, siRNA was transfected into cells first, and we changed the culture medium after 20 h; then, plasmids were transfected, and we harvested cells after 36–48 h. All sequences of siRNAs and shRNAs are shown in Table 1.

### 2.5. RT-qPCR

Total RNA was extracted by TRIzol reagent at a ratio of 1 mL per 1 × 10^6^ cells, and cDNA was obtained by a Vazyme RT kit. RNA was stored at −80 °C, and cDNA was stored at −20 °C temporarily. qPCR was analyzed by a TB Green Premix Ex Taq kit with a two-step qPCR protocol (step 1: 95 °C, 3 min; step 2: 95 °C, 10 s; 60 °C, 30 s, × 39 cycles; step 3: melt curve test). The fluorescence signals were detected and analyzed by the IQ5 sequence detection system (Applied Biosystems, Waltham, MA, USA). All sequences of qPCR are listed in Table 1.

### 2.6. Western Blot and Coimmunoprecipitation

For Western blot, cells were lysed by TNTE buffer (50 mM Tris-HCl, 150 mM NaCl, 0.5% Triton × 100, 1 mM EDTA, 1 mM Na_3_VO_4_, 25 mM NaF, 10 mM Na_4_P_2_O_7_, 10 mM H_2_O, and protease inhibitors (5 mg/mL PMSF, 0.5 mg/mL leupeptin, 0.7 mg/mL pepstatin, and 0.5 mg/mL aprotinin)). After 30 min of incubation on ice, the cell lysates were concentrated by high-speed centrifugation at low temperature (12,000 rpm, 30 min at 4 °C). Proteins were separated by 10% SDS-PAGE electrophoresis at constant voltage (80 V, 20 min; 120 V, 1 h.). Then, the proteins were transferred to nitrocellulose membranes under constant current (125 mA, 1 h 20 min.). Nitrocellulose membranes with different molecular weights (M.W.) of proteins were treated with blocking buffer (5% skimmed milk dissolved in TBST buffer) for 1 h at room temperature. Different M.W. proteins were incubated with related primary antibodies (Table 2) overnight at 4 °C followed by hybridization with secondary antibodies for 2 h at room temperature.

For coimmunoprecipitation, the cells were lysed with RIPA solution and PMSF (1:100) at a ratio of 600 μL per 1 × 10^7^ cells. After 30 min of incubation on ice, the cell lysates were concentrated by high-speed centrifugation at low temperature (12,000 rpm, 30 min at 4 °C). After removing 100 μL of the upper liquid into a new tube as the input group, the remaining lysates were incubated with anti-FLAG magnetic beads and rotated at room temperature for no less than 5 h at room temperature or overnight at 4 °C. The beads were washed with RIPA lysis buffer (500 μL, three times), and the proteins were eluted with 60 μL of glycine-HCl solution (pH = 2–3). The pH of elution was neutralized using 6 μL of 1 M Tris-HCl (pH = 7.5) and 6 μL of 1.5 M NaCl (pH = 7.4). The SDS-PAGE protocol was used as mentioned above to analyze target proteins.

### 2.7. RNA-Seq and ChIP-Seq Analysis

RNA-seq and ChIP-seq data were obtained from the Gene Expression Omnibus (GEO) database GSE120988. Data processing was described previously [23]. Visualization was performed by IGV_2.9.4.

## 3. Results

### 3.1. ASH2L Is Necessary for the Expression of DPY30

**Figure 1:**
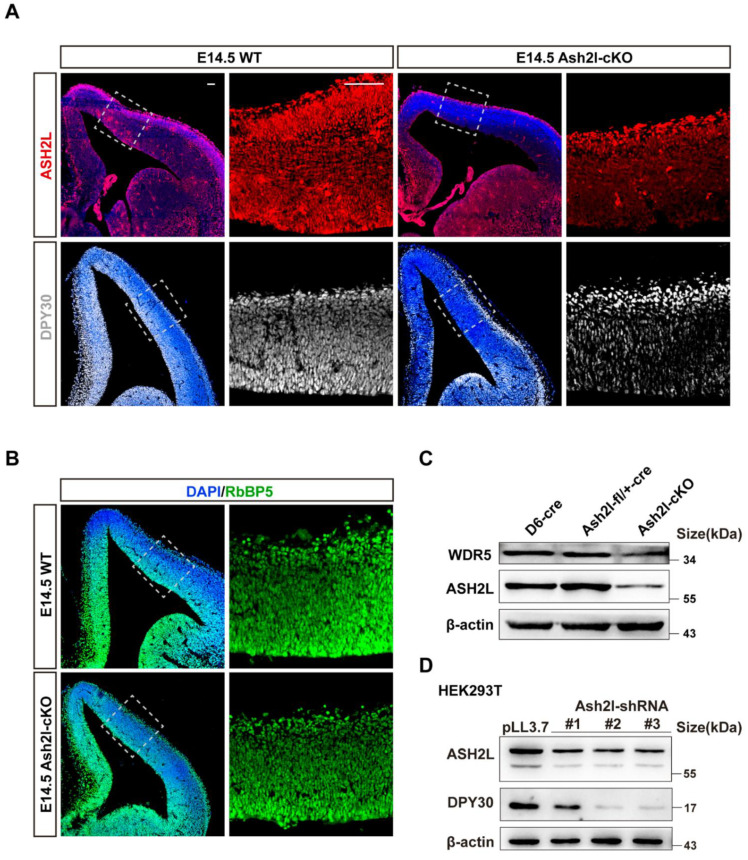
Ash2l is crucial for the expression of DPY30 both in vivo and in vitro. (**A**) Immunofluorescence of ASH2L and DPY30 in the E14.5 dorsal cortex. Scale bars = 100 μm. (**B**) Immunofluorescence of RbBP5 in the E14.5 dorsal neocortex. (**C**) Western blot to detect the expression of ASH2L and WDR5 in the E14.5 neocortex. (**D**) Detection of DPY30 and ASH2L in HEK293T cells transfected with Ash2l-shRNAs, harvested 72 h after transfection. (**C**,**D**) were biologically repeated three times.

The assembly of WARD has been intensively studied for decades, and key amino-acid residues have been identified. However, inter-regulation among these units is not fully understood. Previously, to unravel the function of ASH2L in embryonic neocortical development, we constructed transgenic mice whose Ash2l loci were flanked by the loxP sites [23]. The D6-Cre line was used to conditionally mutate Ash2l in the neocortex [30], and the mutated offspring was termed Ash2l-cKO. Here, we further analyzed these Ash2l-cKO mice and observed the expression of ASH2L, DPY30, and RBBP5 in the E14.5 (Embryonic day 14.5) neocortex (Figure 1A,B). The expression of both ASH2L and DPY30 significantly decreased in Ash2l-cKO mouse brain (Figure S1A). We unexpectedly found that, when ASH2L was depleted, the expression of both DPY30 and RbBP5 decreased concurrently. Additionally, since the WDR5 antibody is not suitable for immunofluorescence, we used extracted proteins from the E14.5 neocortex to detect the expression of WDR5 by Western blot, and there was a slight reduction in WDR5 (Figure 1C). We also observed an extreme loss of H3K4me3 histone modification in the dorsal cortex, while H3K4me1 modification remained unchanged (Figure S1B), which is in line with other reports indicating that the loss of DPY30 uniquely affects H3K4me3, leaving H3K4me1 unaffected [31]. Thus far, we have reported an interesting phenomenon in which the expression of WRD (WDR5, RbBP5, and DPY30) decreases following ASH2L knockout.

Since the reduction in DPY30 was the most obvious, we decided to explore how the DPY30 protein is reduced, and we used the HEK293T cell line (originated from human) as a major platform for mechanistic study, which requires high similarity between mouse and human DPY30. We compared the protein sequences of human and mouse DPY30, and their identity was approximately 97% (Figure S1C). We verified the decrease in DPY30 in HEK293T cell lines (Figure 1D). We then decided to focus on the mutual regulation of DPY30 and ASH2L. We overexpressed ASH2L and found that there was a conspicuous increase in DPY30 (Figure S1D). However, when DPY30 was overexpressed, there was no significant alteration in either ASH2L (Figure S1E). These results indicate that the existence of DPY30 relies on ASH2L. Thus, the following study aimed to explain why DPY30 is significantly decreased after ASH2L depletion.

**Figure 2:**
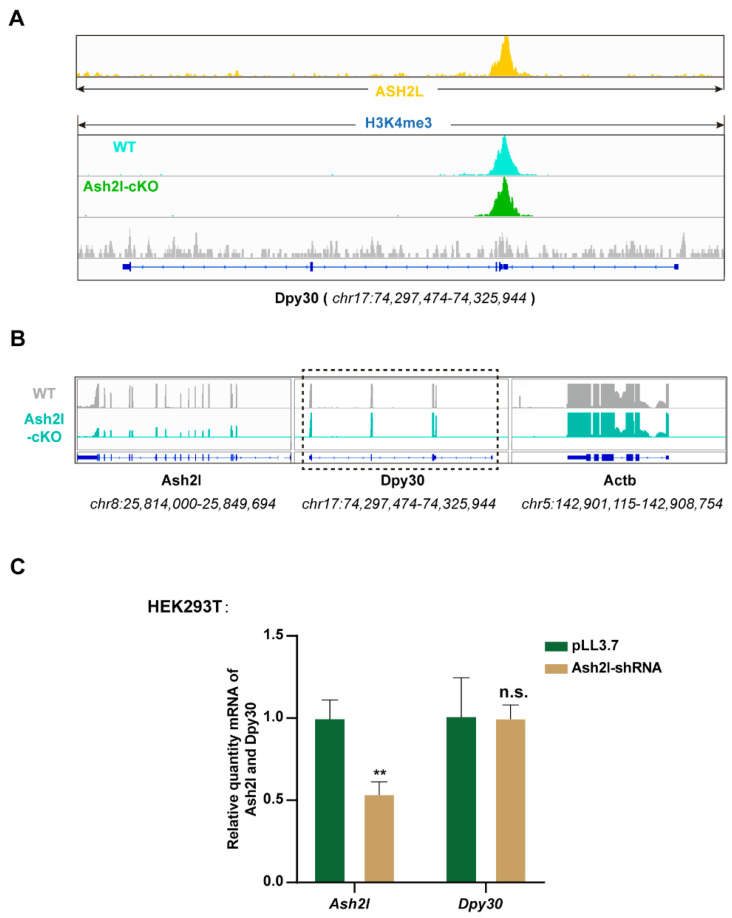
ASH2L does not regulate the transcription of Dpy30. (**A**) H3K4me3 and ASHS2L ChIP-seq visualized by gene track snapshot at Dpy30 gene loci. (**B**) RNA-seq result visualized by gene track snapshot at Ash2l, Dpy30, and Actb gene loci. (**C**) RT-qPCR to detect the expression of DPY30 after Ash2l knocked down in HEK293T cell line. Statistical analysis: ** *p* < 0.01.

**Figure 3:**
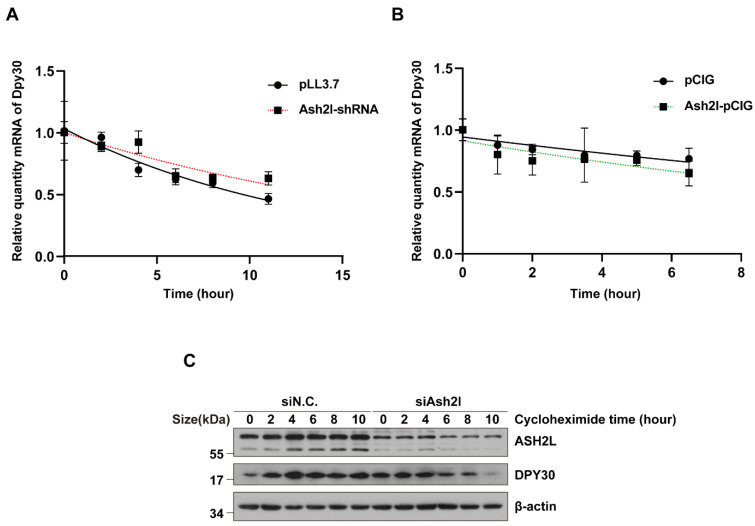
ASH2L is necessary for the stability of DPY30. (**A**,**B**) mRNA stability assays to detect the relative quantity of Dpy30 mRNA by RT-qPCR after knockdown and overexpression of Ash2l followed with actinomycin D (10 μg/mL) treatment. Relative quantity is normalized by GAPDH at each timepoint. The experiment was repeated three times, and the standard deviation is shown. The *t*-test at each timepoint indicates no significant difference. (**C**) HEK293T cells were transfected with Ash2l-shRNAs for 36 h and treated with cycloheximide (50 μg/mL) for the duration shown in hours. The expression levels of ASH2L and DPY30 were tested by Western blotting. The experiment was biologically repeated three times.

Theoretically, the decrease in DPY30 can result from reduced transcription and translation or degradation of mRNA and protein. First, we inspected the ChIP-seq data of H3K4me3 and ASH2L. Although there was direct binding of ASH2L and H3K4me3 at Dpy30 loci, we did not find significant changes in the H3K4me3 peak at the Dpy30 loci, which indicates that the transcription of Dpy30 is independent of ASH2L (Figure 2A). To further verify the hypothesis, we revisited the transcriptome data that we previously generated from both normal and Ash2l-cKO neural stem cells [23]. Indeed, we found that the transcription of Dpy30 did not change obviously in Ash2l-cKO neural stem cells (Figure 2B). We also performed a knockdown experiment on the HEK293T cell line, and Ash2l-KD had no significant influence in the expression level of Dpy30 mRNA (Figure 2C). Thus, we further investigated the stability of Dpy30 mRNA. We used actinomycin D to inhibit transcription globally after the overexpression or knockdown of Ash2l, and we consecutively detected the quantity of Dpy30 mRNA. The overexpression and knockdown were validated, and it turned out that the stability of Dpy30 mRNA is not affected by the perturbation of Ash2l (Figure 3A,B and Figure S2A). We then speculated that the protein stability of DPY30 can be more predominantly affected by ASH2L, instead of the transcription. To focus on the stability of DPY30 protein, we inhibited protein translation using cycloheximide after the knockdown of Ash2l and then consecutively detected DPY30. In control cells, ASH2L was stable enough to exist for more than 10 h without obvious degradation, and DPY30 was accordingly also stable with fluctuation. However, when ASH2L was partially depleted, there was a conspicuous decrease in DPY30 (Figure 3C). In conclusion, we believe that the stability of DPY30 is compromised after Ash2l knockdown.

### 3.2. The Stability of DPY30 Relies on Direct Binding with ASH2L

**Figure 4:**
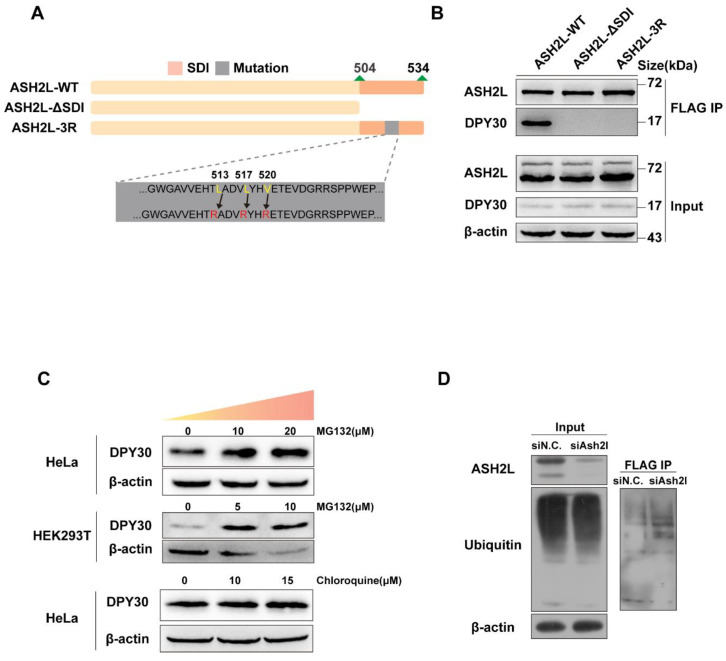
The stability and degradation of DPY30 (**A**) Construction of the ASH2L-WT, ASH2L-ΔSDI, and ASH2L-3R mutations. (**B**) Coimmunoprecipitation assay using FALG-tagged ASH2L-WT, ASH2L-SDI, and ASH2L-3R to detect their binding with DPY30. (**C**) Gradient doses of MG132 (upper panel) and chloroquine (lower panel) were used to inhibit the cell proteasome and lysosome, respectively, and the relative expression level of DPY30 in different cell lines was observed by Western blot. Experiments in different cell lines were repeated three times. (**D**) Coimmunoprecipitation assay using FLAG-tagged DPY30 to detect the binding of ubiquitin after knockdown of ASH2L.

The abovementioned results indicate the instability of the DPY30 protein after the loss of ASH2L. It is highly likely that the direct binding of ASH2L and DPY30 reinforces the stability of DPY30. Previously, Park et al. reported the structure of the WRAD complex [32], and Chen et al. proved that the SDI domain at the C-terminus of ASH2L—more precisely, three residues, L513/L517/V520, within the SDI domain—is responsible for the binding of DPY30 to ASH2L (Figure 4A) [10], and mutating L513/L517/V520 to R513/R517/R520 would disable the binding of DPY30. Here, we also validated that the three residues are important for the binding of DPY30, as we used FLAG-tagged wildtype ASH2L (ASH2L-WT) and two mutated forms of ASH2L (ASH2L-ΔSDI and ASH2L-3R) to immunoprecipitate other components of WRAD. It is clear that ASH2L-WT could precipitate DPY30, while ASH2L-ΔSDI and ASH2L-3R were not able to coprecipitate DPY30 (Figure 4B). In addition, neither mutated form of ASH2L affected the binding of WDR5 and RbBP5 (Figure S2B).

### 3.3. The Degradation of DPY30 Relies on the Ubiquitin-Proteasome System

Now that we confirmed the degradation of DPY30 protein after ASH2L depletion, we next decided to determine how the protein is degraded. Generally, both the ubiquitin-proteasome system and the lysosome system can be responsible for DPY30 degradation [33]. MG132 is able to competitively inhibit the proteolytic activity of proteasomes and, therefore, prevents the degradation of target proteins modified with ubiquitin [34]. We initially applied MG132 to inhibit the ubiquitin-proteasome system and further detected the protein level of DPY30 (Figure 4C, upper panel). After inhibition of the ubiquitin-proteasome system by increasing the concentration of MG132, there was a significant increase in DPY30 with the increase in concentration. In parallel, we used chloroquine to inhibit the lysosomal system and noticed that there was no significant increase in DPY30 (Figure 4C, lower panel). As it was indicated that DPY30 degrades through the proteasome system, more direct evidence could be provided by coimmunoprecipitation with ubiquitin. We constructed a plasmid that expressed DPY30 tagged with FLAG. We transfected cells with siAsh2l, followed by transfection of FLAG-tagged DPY30 and exogenous ubiquitin. Although there was no obvious increase in overall ubiquitin precipitated by FLAG-tagged DPY30 (Figure 4D, left), ubiquitin that was directly precipitated by a FLAG-tagged DPY30 increased (Figure 4D, right). Although the ubiquitination site of DPY30 has not been reported before, we used UbiNet, a web resource offering prediction of the protein ubiquitination site to predict potential sites [35] (Figure S2C). Therefore, we can conclude that DPY30 is degraded primarily via the ubiquitin-proteasome system.

### 3.4. DPY30 Is Capable of Ameliorating the Outcome of ASH2L Depletion

**Figure 5:**
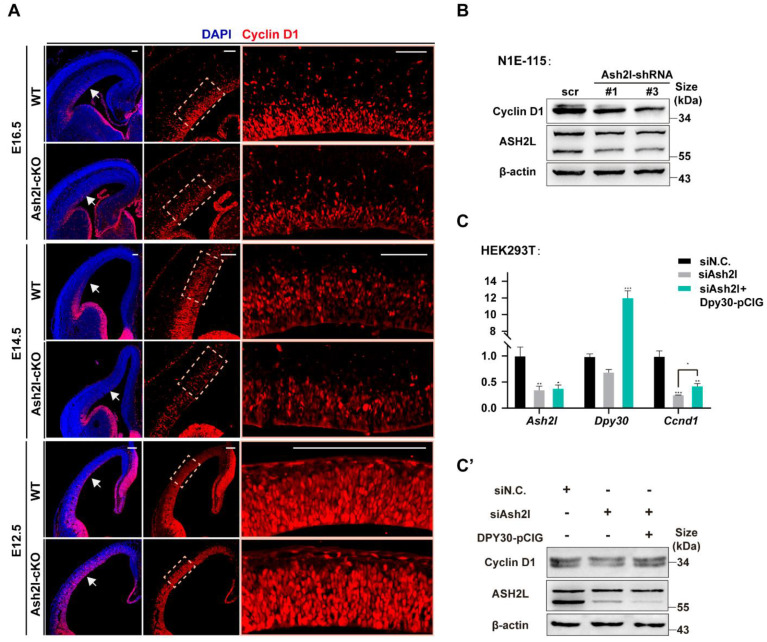
DPY30 ameliorates the outcome of Ash2l depletion (**A**) Immunofluorescence of Cyclin D1 in the dorsal neocortex of WT and Ash2l-cKO mice at E12.5, E14.5, and E16.5. (**B**) Ash2l-shRNA was used to interfere with the expression of Ash2l in the N1E-115 cell line, and the expression of Cyclin D1 was decreased compared to that of the control group. (**C**) RT-qPCR to detect the expression of Ccnd1 after overexpression of DPY30. The siAsh2l group and siAsh2l + Dpy30-pCIG group were compared with yjr siN.C. group. Statistical analysis: * *p* < 0.05; ** *p* < 0.01; *** *p* < 0.001. (**C’**) Western blotting was biologically repeated three times and applied to detect Cyclin D1 protein after the overexpression of DPY30.

A previous study showed the involvement of DPY3 in chromatin regulators other than COMPASS [36]. Here, we found that the overexpression of DPY30 can partially rescue the phenotype of ASH2L knockdown. One key phenotype of Ash2l-cKO mice is the abnormal cell cycle, which is stagnated at the G1 phase, with a reduction in the G1-to-S checkpoint Cyclin D1 (encoded by Ccnd1) [23]. We detected the expression of Cyclin D1 in both E14.5 and E16.5 dorsal telencephalon and observed a significant reduction (Figure 5A and Figure S2D). Consistent with this, we also found that Cyclin D1 was downregulated when Ash2l expression was interfered with by shRNAs in cell lines (Figure 5B). When we overexpressed DPY30 in ASH2L knockdown cells, we observed that both mRNA and protein levels of Ccnd1 were elevated to normal levels (Figure 5C,C’). Thus, we found that DPY30 can function to activate Ccnd1 expression independent of ASH2L, indicating multiple functionalities of DPY30 in the regulation of gene expression, such as recruiting other regulative complexes.

## 4. Discussion

COMPASS is responsible for generating H3K4me1/me2/me3 modifications, and the enzymatic activity relies on the assembly of methyltransferase and WRAD. Although many studies have explained the assembly of COMPASS, the mutual regulation among WRAD factors was partially missed by others. It is intriguing that we found that the existence of Dpy30 is dependent on Ash2l both in vivo and in vitro. Specifically, we proved that the stability of the DPY30 protein, rather than its transcription and translation, is dependent on its binding with ASH2L.

In the current study, we observed the degradation of DPY30 by the ubiquitin-proteasome system, while there remain several interesting areas for further investigation, including the determination of the ubiquitination site of DPY30 and the E3 ligases that are responsible for the degradation. The verification of ubiquitination site can be achieved by mutation studies, while determining E3 ligases may require more effort, considering that the human proteome codifies for more than 600 E3s that can be categorized into three major groups, homologous to the E6AP carboxyl terminus (HECT), really interesting new gene (RING), and RING between RING (RBR) [37], and the ubiquitination of DPY30 can be achieved by any one the three groups of E3s. A proteomics study is necessary after the coimmunoprecipitation of DPY30 and its binding proteins, and we may expect to discover the corresponding E3 ligases and the modification of both E3 ligase and DPY30, which may be required for the substrate recognition of E3s [38]. Considering the importance of DPY30 in development and cancers, our discovery is necessary to explain how DPY30 stably exists in cells.

Firstly, stable DPY30 relies on its binding with ASH2L. We observed a dramatic decrease in DPY30 after Ash2l depletion, and we proved that the stability of DPY30 relies on binding with ASH2L. Specifically, we found the three amino-acid residues (L513/L517/V520) in the SDI domain of ASH2L that play vital roles in binding DPY30, consistent with previous research [10,39], as we noticed that the ASH2L-3R mutant could block the binding site of DPY30.

Secondly, DPY30 rescues the decreased expression of Ccnd1 in Ash2l-depleted cells. A previous study indicated that the depletion of Ash2l in neural progenitors causes cell-cycle arrest with a significant decrease in Ccnd1 (Cyclin D1) [23]. In this study, we found that Ccnd1 expression could be rescued by Dpy30 in Ash2l-depleted cells (Figure 5C,C’), which indicates that DPY30 can function independently of WRAD, at least partially. Notably, WRAD complex proteins appear to have separate functions beyond their role within COMPASS complexes. In addition, structural analysis of DPY30 revealed that the D/D domain of DPY30 can not only bind ASH2L, but also recognize BAP18 [12], a component of the nucleosome remodeling complex (NURF), which is regarded as an epigenetic reader of H3K4 trimethylation [40,41,42]. Thus, it is possible that DPY30 can recruit NURF to activate Ccnd1 expression. Recently, researchers hypothesized that COMPASS and NURF, two epigenetic writers, may coordinate with each other to regulate the expression of target genes [13,36]. Thus, along with the participation of COMPASS, it is possible that DPY30 binds BAP18 to enroll another epigenetic pathway of NURF [12].

Lastly, most epigenetic regulation, especially histone-modifying enzymes, exists in the form of complexes, and each subunit performs a different function so that it can achieve well-organized regulation. There are indicative networks of interactions between the different subunits. This study found that there is an interdependent relationship between different subunits, providing a new perspective for understanding the functional regulation of such complexes.

## Figures and Tables

**Table 1:** Primer sequences used in this article (“h” denotes human sequence, “m” denotes mouse sequence.).

Name	Sequence
hAsh2l-F	ATGGATACTCAGGCGGGCTC
hAsh2l-R	TCAGGGTTCCCATGGGGG
hAsh2l-ΔSDI-F	ATGGATACTCAGGCGGGCTC
hAsh2l-ΔSDI-R	CATGTCACTCATAGGGCGGTAA
hAsh2l-3E-F	ACCCTTGCTGACGTCCTCTATCACCTCGAGACAGAAGTGGATGGGAGGCG
hAsh2l-3E-R	CTCGAGGTGATAGAGGACGTCAGCAAGGGTGTGCTCTACCACGGCGCC
Ash2l-qPCR-F	CCTACTTTCTCCGGAAGCAAG
Ash2l-qPCR-R	GACAATGTTATTGGGCCAAGTC
Dpy30-qPCR-F	AACGCAGGTTGCAGA AAATCCT
Dpy30-qPCR-R	TCTGATCCAGGTAGGCACGAG
Ccnd1-qPCR-F	CTGATTGGACAGGCATGGGT
Ccnd1-qPCR-R	GTGCCTGGAAGTCAACGGTA
Gapdh-qPCR-F	GGTCATCCATGACAACTTTGG
Gapdh-qPCR-R	GGCCATCACGCCACAG
siAsh2l-1#	CAGTAAAGATCCAGAAGAA
siAsh2l-2#	GGTGCCTGGTATTTTGAAA
siAsh2l-3#	CGAAGACAATGTTCTCCAA
mAsh2l-shRNA-1	CAAGAAGGCCAGAAGTGATCCTTTATTCAAGAGATAAAGGATCACTTCTGGCCTTCTTGTTTTTT
mAsh2l-shRNA-3	CCTGTGTCTGTGTGTTCCAAATTCAAGAGATTTGGAACACACAGACACAGGTTTTTT
hAsh2l-shRNA-1	CCGGGTGACTTGTTATCCTACTATACTCGAGTATAGTAGGATAACAAGTCACTTTTT
hAsh2l-shRNA-2	CCGGCCGAAGACAATGTTCTCCAAACTCGAGTTTGGAGAACATTGTCTTCGGTTTTT
hAsh2l-shRNA-3	CCGGCCTGCTTGTATGAACGGGTTTCTCGAGAAACCCGTTCATACAAGCAGGTTTTT

**Table 2:** Antibodies used in the study.

Name	Resource
Anti-Ash2	BETHYL (Waltham, MA, USA, #A300–112A)
Anti-DPY30	Sigma (St. Louis, MO, USA, #HPA043761)
Anti-WDR5	R&D system (Minneapolis, MN, USA, #AF5810)
Anti-RbBP5	Abcam (Cambridge, UK, #ab52084)
Anti-Cyclin D1	Abcam (Cambridge, UK, #ab16663)
Anti-FLAG	ABclonal (Cambridge, UK, #WH166500)

## Data Availability

Not applicable.

## Supplementary Materials

The following supporting information can be downloaded at https://www.mdpi.com/article/10.3390/cells11091450/s1: Figure S1. Ash2l is important for H3K4me3 and DPY30, and the sequence of DPY30 is highly conserved between species; Figure S2. The interaction among WRAD components and the ubiquitination of DPY30.
