# Supplementary material for: The Ash2l SDI Domain Is Required to Maintain the Stability and Binding of DPY30
Source: Cells. 2022 Apr 25;11(9):1450. doi: 10.3390/cells11091450 (PMC9103646; doi:10.3390/cells11091450)
Supplement: Supplementary file 1 [file cells-11-01450-s001.zip › cells-1648149-supplementary.pdf]

Supplementary materials

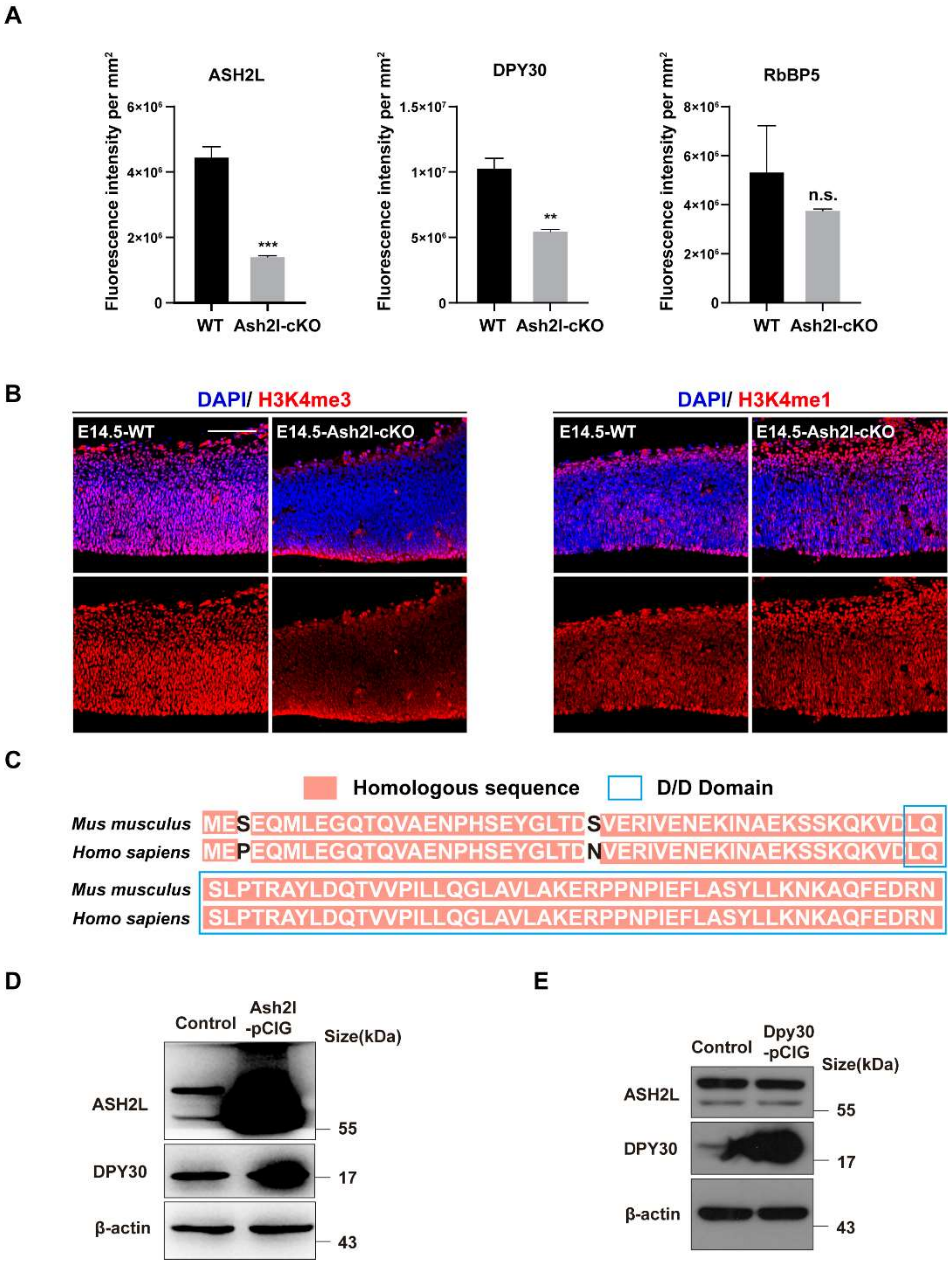

Figure S1. Ash2l is important for H3K4me3 and DPY30, and the sequence of DPY30 is highly conserved between species. (A) Quantitative analysis of ASH2L, DPY30 and RbBP5 fluorescence intensity of E14.5 mouse brain staining. Statistical analysis: "*", p<0.05; "**" p<0.005; "***", p<0.0005. (B) Immunofluorescence of H3K4me3 and H3K4me1 in the WT and Ash2l-cKO E14.5 dorsal cortex. (C) Protein homology analysis of DPY30 between *Mus musculus* and *Homo sapiens*. (D) and (E) HEK293T cells were transfected with pCIG-ASH2L and pCIG-DPY30 separately, and the expressions of ASH2L and DPY30 were tested by Western blotting.

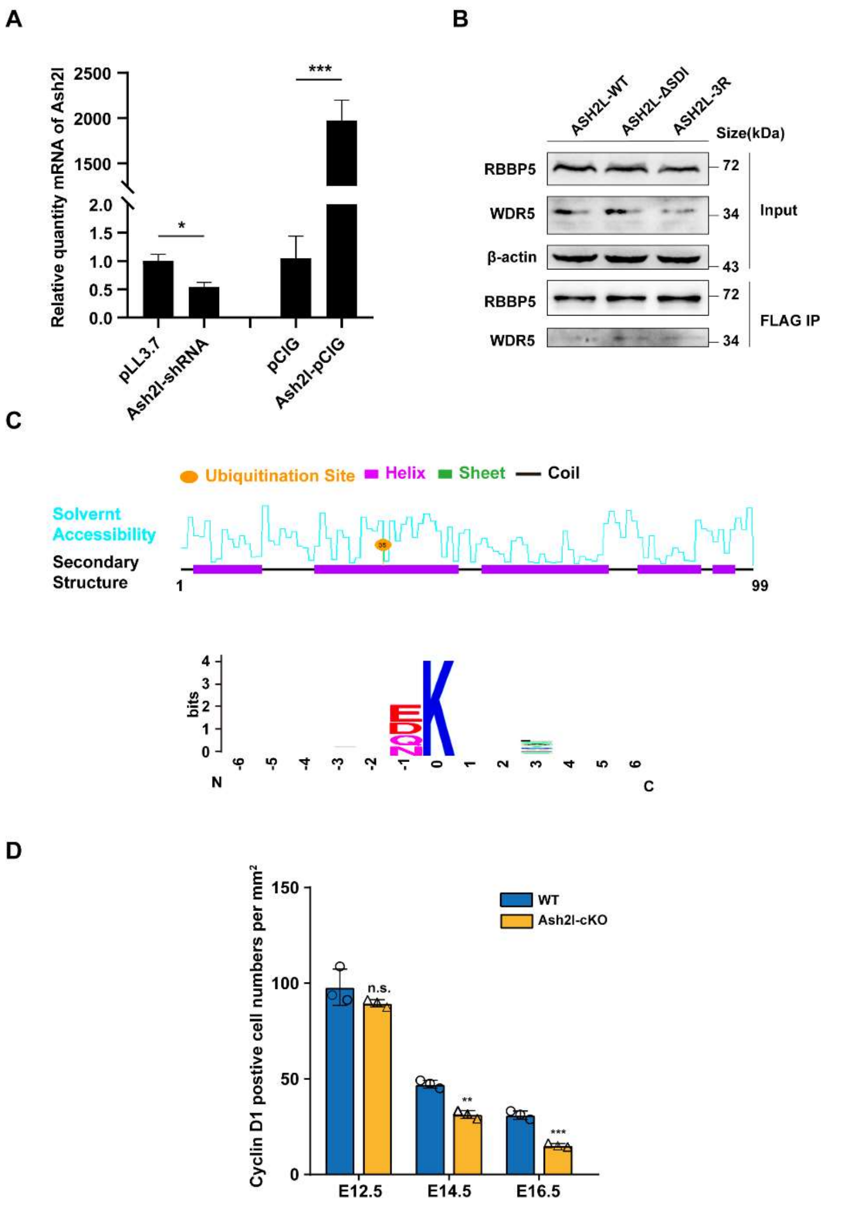

Figure S2. The interaction among WRAD components and the ubiquitination of DPY30 (A) RT-qPCR to validate the overexpression and knockdown of Ash2l. (B) Coimmunoprecipitation assay using ASH2L-WT, ASH2L-ΔSDI and ASH2L-3R constructs to demonstrate the binding of ASH2L-RbBP5 and ASH2L-WDR5 won't be disturbed by ASH2L mutations. (C) Prediction of the ubiquitination site on DPY30 by the UbiNet website. (D) Quantitative analysis of Cyclin D1 positive cell numbers of E12.5, E14.5 and E16.5. Statistical analysis: "*", $p<0.05$; "**" $p<0.005$; "***", $p<0.0005$.
